# Overexpression of the *OsHY5L2* Alters the Fine Structure and Physicochemical Properties of Endosperm Starch in Rice (*Oryza sativa* L.)

**DOI:** 10.3390/plants14182888

**Published:** 2025-09-17

**Authors:** Yuan Wu, Mingyang Zeng, Junhao Zhang, Haiyan Jiang, Lixia Ma, Dong Liu, Yongjun Zeng

**Affiliations:** Ministry of Education Key Laboratory of Crop Physiology, Ecology and Genetic Breeding, Jiangxi Agricultural University, Nanchang 330045, China; wy11202122@163.com (Y.W.); myzengbio@163.com (M.Z.); zjh3140712@126.com (J.Z.); haiyan200228@163.com (H.J.); malxjxau@126.com (L.M.)

**Keywords:** rice, *OsHY5L2*, starch metabolism, fine structure, physicochemical properties

## Abstract

Although the role of *OsHY5L2* in promoting photomorphogenic development is well characterized, its function in regulating rice quality is poorly understood. In this study, we found that *OsHY5L2* plays an important role in regulating starch metabolism and modulating its fine structure and physicochemical properties. Overexpression of *OsHY5L2* significantly reduced the chalky grain rate and degree of chalkiness but dramatically increased the head rice rate. *OsHY5L2* was found to negatively regulate the accumulation of starch in rice endosperm by inhibiting starch biosynthesis and promoting starch hydrolysis. Transcriptomic analysis revealed that *OsHY5L2* mainly regulated the expression of genes encoding enzymes involved in starch and sucrose metabolism. Moreover, *OsHY5L2* overexpression induced the formation of numerous pinhole structures on the surfaces of starch granules. Analysis of the amylopectin chain length distribution showed that overexpression of *OsHY5L2* decreased the proportion of ultra-short chains (DP 6–7) and intermediate chains (DP 13–24) of amylopectin while increasing the proportion of short chains (DP 8–12) and long chains (DP 25–36). Further studies demonstrate that *OsHY5L2* overexpression altered the pasting properties of rice starch by affecting its multi-level structure and function. The results of this study improve our understanding of the functions of *OsHY5L2* in regulating rice quality.

## 1. Introduction

Rice is one of the most important food crops and serves as the main carbohydrate source for nearly half of the global population, with approximately 500 million tons of milled rice produced annually [1]. Along with changes in the dietary habits of people associated with improvements in their economic situation, the demand for high-quality rice has increased. Therefore, understanding the mechanisms by which high-quality rice characteristics are formed is critical for the sustainable development of the rice industry.

Rice quality is determined by a combination of traits affecting appearance, processing characteristics, eating and cooking quality (ECQ), and nutrition. Among these, ECQ is considered the primary concern for the average consumer and, as such, determines market competitiveness [2]. The endosperm is the primary edible portion of the rice grain which is composed predominantly of amylose and amylopectin. Rice with low amylose content (AC) tends to have high stickiness and adhesiveness, improving its ECQ [3]. Amylopectin is the primary component of rice starch, which determines both the hierarchical structure and physicochemical properties of starch. Amylopectin is also the main driver of the significant differences in ECQ between rice varieties with similar AC [4]. According to their degree of polymerization (DP), amylopectin branch chains are generally classified into four types: A chain (DP 6–12), B1 chain (DP 13–24), B2 chain (DP 25–36), and B3 chain (DP ≥37) [5]. The chain-length distribution of amylopectin is closely associated with the ECQ of rice. For instance, the enzyme encoded by the *SSSI* gene is responsible for the biosynthesis of short-chain amylopectin, while the enzymes encoded by the *SSSIIa* and *SSSIIb* genes are responsible for the biosynthesis of intermediate- and long-chain amylopectin [6]. The downregulation of *SSSIIa* or loss of *SSSIIb* decreases the proportion of intermediate- and long-chain amylopectin while increasing the proportion of short-chain amylopectin, leading to improved grain ECQ [6,7,8]. SBE facilitates the branching of amylose to form amylopectin. The enzyme encoded by the *SBEI* gene is responsible for the biosynthesis of intermediate- and long-chain amylopectin. Loss of function in the *SBEI* gene reduces the proportion of intermediate- and long-chain amylopectin while increasing the proportion of short-chain amylopectin, also resulting in improved grain ECQ [9].

Rice quality is influenced by a combination of genetic factors, environmental signals, and cultivation conditions. Environmental signals such as temperature, light, and water availability affect the quality of rice by modulating both AC and amylopectin fine structures [10]. Although light conditions encompass many variables, the majority of studies to date have evaluated the effect of light intensity on rice quality. For example, exposure to low light significantly increases both the chalky grain rate and chalkiness degree but dramatically reduces the rates of brown rice, milled rice, and head rice [11,12]. Low-light-driven chalkiness is primarily attributed to insufficient assimilate supply, resulting in poor grain filling and poor starch granule development in the endosperm [13]. In addition, several studies have demonstrated that exposure to low light significantly decreases the breakdown viscosity (BDV) and peak viscosity (PKV) of endosperm starch while increasing the setback viscosity (SBV), thereby adversely affecting rice ECQ [11,12,14]. Low light stress appears to influence rice quality primarily by reducing the AC and altering the chain length distribution of amylopectin [15]. Although research has clearly illustrated that light intensity affects rice quality, the mechanism by which light signals regulate rice quality has not yet been fully elucidated. Moreover, the rice quality-related functions of genes encoding key components of the light signaling pathway have not yet been explored.

Numerous transcription factors have been reported to play important roles in the regulation of starch biosynthesis in rice endosperm by directly activating or inhibiting the expression of starch biosynthesis-related genes. The basic leucine zipper (bZIP) transcription factor OsbZIP60/OPAQUE3 positively regulates the expression of several starch biosynthesis-related genes such as *GBSSI*, *AGPL2*, *SBEI*, and *ISA2*. The knockout mutant *o3* had a lower grain thickness with reduced starch content and altered starch morphology when compared with the wild-type [16]. The bZIP transcription factor OsbZIP10 can also directly bind to the promoters of genes crucial to starch biosynthesis, including *OsGIF1* and *OsAGPS1*, and genes pivotal to ROS homeostasis, such as *OsAPX1*, *OsPRX47*, and *OsCAT2*. Further studies revealed that the influence of OsbZIP10 on starch biosynthesis is closely associated with redox homeostasis which, in turn, is important for the proper functioning of OsGIF1 and OsAGPS1 [17]. Similarly, other transcription factors (NAC, MYB, and MADS) have been reported to directly regulate the expression of starch biosynthesis genes in rice endosperm. *OsNAC24* is highly expressed in developing endosperm and can interact with OsNAP to form a transcription complex that directly activates *OsGBSSI* and *OsSBEI* expression, thereby regulating starch biosynthesis in the rice endosperm [18]. OsMADS14 can directly bind to the CArG boxes of *OsAGPL2* and *GBSSI* to activate their expression, regulating the synthesis of storage starch during the process of grain filling in rice [19]. Rice *OsMYB73* is mainly expressed in early-developing pericarp and endosperm. The knockout mutant of *myb73* had longer grains with increased amylose content and more strongly altered physicochemical properties of starch, when compared with the wild-type. Further studies have revealed that OsMYB73 interacts with OsNF-YB1 and binds to *OsISA2* and *OsYUC11* to regulate endosperm starch accumulation [20].

The *ELONGATED HYPOCOTYL5* (*HY5*) gene encodes a bZIP transcription factor and plays a positive role in light signaling [21]. This transcription factor can activate the transcription of many light-responsive genes by directly binding to their promoters [22]. *HY5* was originally identified as a key gene regulating photomorphogenesis in *Arabidopsis thaliana* [23]. Loss-of-function *HY5* alleles displayed an etiolated phenotype typified by elongated hypocotyls under all light conditions, indicating that *HY5* functions downstream of all photoreceptors to promote photomorphogenesis [21,23]. Further research has revealed that *HY5* also regulates lateral root development, anthocyanin biosynthesis, and stress responses [21]. Consistent with its diverse biological functions, AtHY5 was shown to target 3195 to 11,797 Arabidopsis genes in several previous studies [22,24,25,26]. Although the functions of *HY5* have been extensively studied in dicots, research in monocots remains limited. The amino acid sequence of rice *OsHY5L2* is highly homologous to that of AtHY5. The heterologous expression of *OsHY5L2* can compensate for the photomorphogenic defects of *Arabidopsis HY5* mutants, suggesting that *HY5* plays a conserved role in regulating photomorphogenesis [27]. Moreover, transgenic rice lines overexpressing *OsHY5L2* exhibit photomorphogenesis-related phenotypes such as dwarfism, deep green leaves, and shortened internodes, whereas the knockout or knockdown of *OsHY5L2* results in lethality at a very early developmental stage [27]. As mentioned above, light serves as an environmental signal regulating rice quality, and *HY5* is a central positive regulator in the light signaling pathway. Therefore, it is necessary to explore whether *OsHY5L2* plays an important role in regulating rice quality by modulating the metabolism and multi-level structure of starch.

The main objectives of this study were to evaluate the effects of *OsHY5L2* overexpression on (a) rice starch metabolism, (b) the fine structural properties of rice starch, and (c) the physicochemical properties of rice starch. The results of this study improve our understanding of the functions of *OsHY5L2* in regulating starch metabolism and modulating its fine structure and physicochemical properties.

## 2. Results

### 2.1. Appearance Quality, Processing Quality, and Starch Content

**Figure 1:**
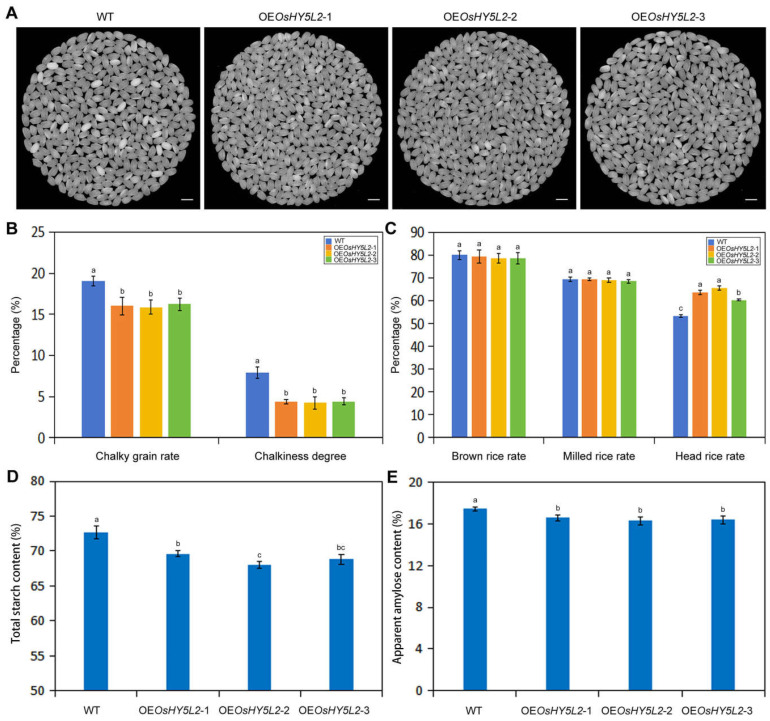
Overexpression of *OsHY5L2* improves rice processing quality and appearance. (**A**) Appearances of milled rice of *OsHY5L2*-overexpressing lines (OE*OsHY5L2*-1, OE*OsHY5L2*-2, and OE*OsHY5L2*-3) and wild-type (WT) Nipponbare. Scale bar = 5 mm. (**B**) Chalky grain rate and degree of chalkiness. (**C**) Brown rice rate, milled rice rate, and head rice rate. (**D**) Total starch content. (**E**) Apparent amylose content. Error bars = SD. Mean value followed by different letters are significantly different (*p* < 0.05).

To explore the function of the *OsHY5L2* gene in regulating rice quality, we first compared the appearance of rice grains produced by the WT and three *OsHY5L2*-overexpressing lines (OE*OsHY5L2*-1, OE*OsHY5L2*-2, and OE*OsHY5L2*-3). Notably, overexpression of *OsHY5L2* significantly reduced the chalky grain rate and degree of chalkiness (Figure 1A,B). Regarding processing quality, while overexpression of *OsHY5L2* did not significantly affect the brown rice rate or the milled rice rate, it noticeably increased the head rice rate (Figure 1C). Moreover, overexpression of *OsHY5L2* significantly reduced the total starch content and apparent amylose content (AAC, Figure 1D,E). These findings suggest that *OsHY5L2* plays a positive regulatory role in improving the appearance and processing quality of rice.

### 2.2. Activity of Starch Biosynthesis-Related Enzymes

**Figure 2:**
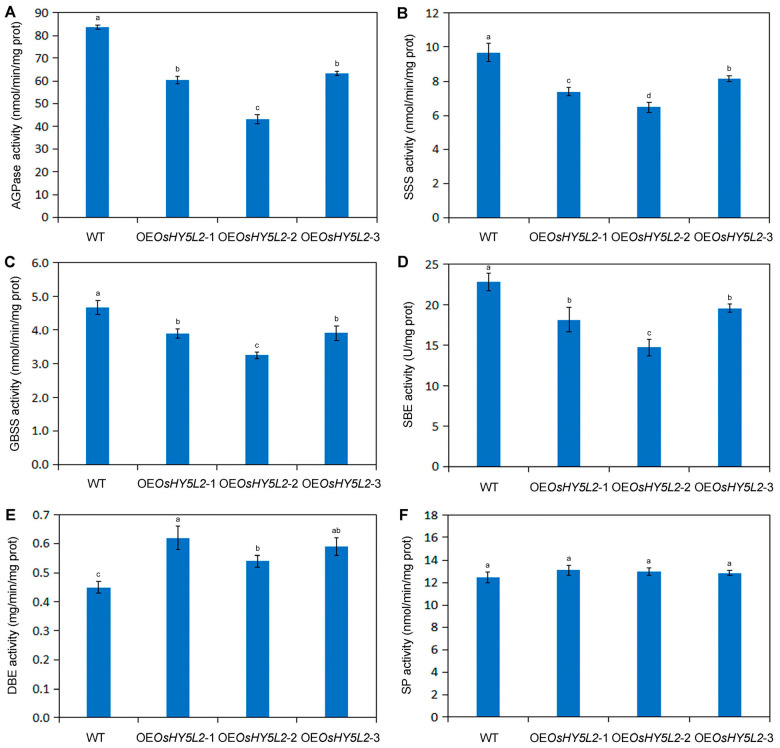
Activities of starch biosynthesis-related enzymes in the developing grains of *OsHY5L2*-overexpressing lines (OE*OsHY5L2*-1, OE*OsHY5L2*-2, and OE*OsHY5L2*-3) and wild-type (WT) Nipponbare. (**A**) AGPase; (**B**) SSS; (**C**) GBSS; (**D**) SBE; (**E**) DBE; (**F**) SP. Error bars = SD. Mean value followed by different letters are significantly different (*p* < 0.05).

Because *OsHY5L2* overexpression was observed to significantly reduce the total starch content and AAC in the rice endosperm (Figure 1D,E), the activities of several starch biosynthesis-related enzymes were evaluated, including AGPase, GBSS, SSS, SBE, DBE, and SP. The results are shown in Figure 2A–E. Compared to the WT, the activities of AGPase, GBSS, SSS, SBE, and DBE were significantly altered in the three *OsHY5L2*-overexpressing (*OsHY5L2*-OE) transgenic lines (Figure 2). Specifically, the activities of AGPase, GBSS, SSS, and SBE were significantly decreased, while DBE activity was markedly increased, in these *OsHY5L2*-OE lines (Figure 2A–E).

### 2.3. Activity of Starch Hydrolysis-Related Enzymes and Levels of Endogenous Hormones

**Figure 3:**
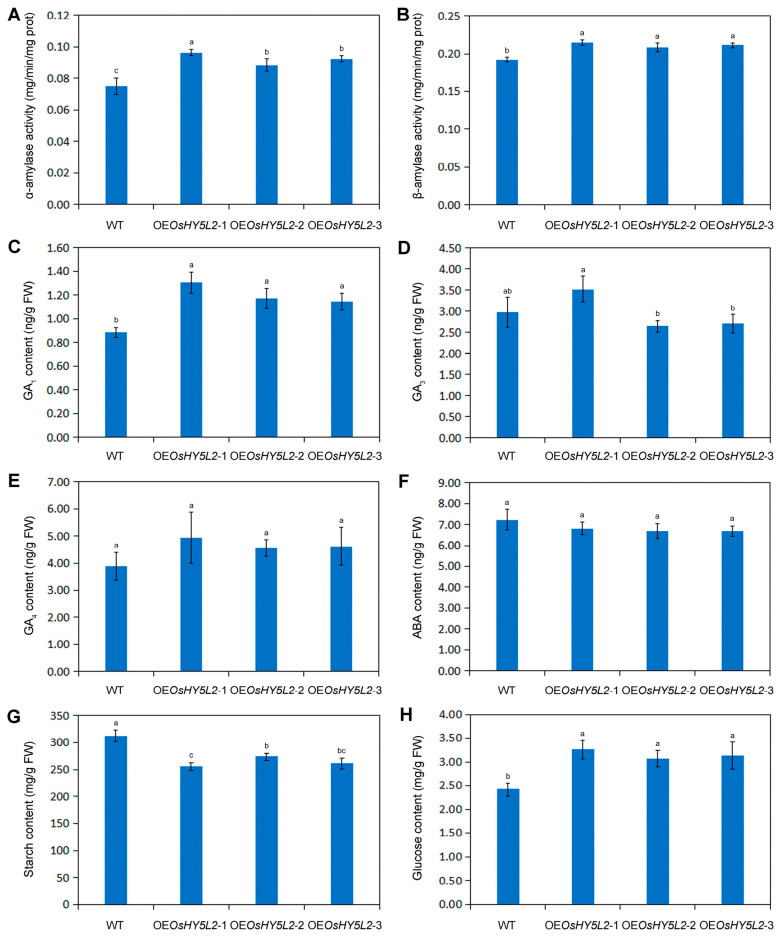
Physiological parameters associated with starch hydrolysis in the developing grains of OsHY5L2-overexpressing lines (OE*OsHY5L2*-1, OE*OsHY5L2*-2, and OE*OsHY5L2*-3) and wild-type (WT) Nipponbare. (**A**) α-Amylase activity; (**B**) β-Amylase activity; (**C**) GA_1_ content; (**D**) GA_3_ content; (**E**) GA_4_ content; (**F**) ABA content; (**G**) Starch content; (**H**) Glucose content. Error bars = SD. Mean value followed by different letters are significantly different (*p* < 0.05).

The activities of both α-AMY and β-AMY were significantly higher in *OsHY5L2*-OE grains than in the WT (Figure 3A,B). As phytohormones such as gibberellins (GAs) and abscisic acid (ABA) regulate starch hydrolysis in cereal endosperm, the contents of these phytohormones were further determined. It was observed that overexpression of *OsHY5L2* resulted in a significant increase in GA_1_ levels in the grains of *OsHY5L2*-overexpressing lines, while the levels of GA_3_, GA_4_, and ABA remained unchanged (Figure 3C–F; GA_7_ was undetectable due to its low concentration). Moreover, overexpression of *OsHY5L2* resulted in a notable decrease in starch content and a significant increase in glucose content in *OsHY5L2*-OE grains (Figure 3G,H). These results indicate that *OsHY5L2* regulates starch hydrolysis by inducing GA_1_ biosynthesis.

### 2.4. Transcriptomic Analysis of Genes Involved in Starch and Sucrose Metabolism

A total of 1012 genes were differentially expressed in *OsHY5L2*-OE grains relative to WT grains, including 736 upregulated DEGs and 276 downregulated DEGs (Tables S1 and S2). According to gene ontology (GO) classification analysis, these DEGs were categorized into 17 biological processes, 3 cellular components, and 11 molecular functions (Figure S1). A Kyoto encyclopedia of genes and genomes (KEGG) enrichment analysis revealed that these DEGs were mainly enriched in pathways such as starch and sucrose metabolism, phenylpropanoid biosynthesis, amino acid biosynthesis, carbon metabolism, and amino sugar and nucleotide sugar metabolism, with starch and sucrose metabolism being the most enriched pathway (Figure S2). Because altered starch and sucrose metabolism has a significant impact on rice quality, we subsequently studied the expression of genes involved in this pathway.

**Figure 4:**
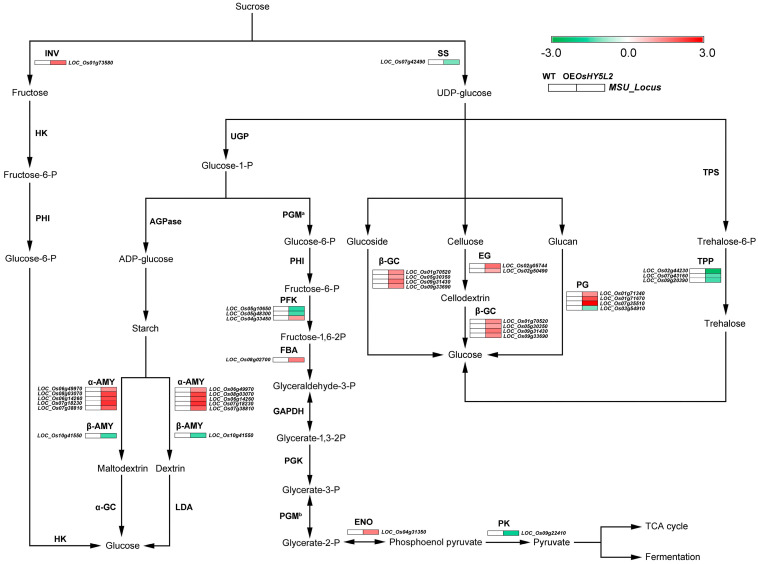
Heatmap of DEGs involved in starch and sucrose metabolism in the developing grains of *OsHY5L2*-overexpressing line (OE*OsHY5L2*-1) and wild-type (WT) Nipponbare. The color gradient indicates the relative expression levels of DEGs from low (green indicates downregulation) to high (red indicates upregulation).

It was observed that overexpression of *OsHY5L2* resulted in the upregulated expression of the gene encoding INV but the downregulated expression of the gene encoding SS (Figure 4 and Table S3). Among the DEGs involved in starch hydrolysis, five genes encoding α-AMY (or its isoenzymes) were upregulated, whereas one gene encoding β-AMY was downregulated (Figure 4 and Table S3). Moreover, overexpression of *OsHY5L2* altered the expression of genes encoding enzymes involved in glycolysis, including three upregulated and three downregulated genes. Specifically, two DEGs encoding phosphofructokinase (PFK) were downregulated and one was upregulated; two DEGs encoding fructose-1,6-bisphosphate aldolase (FBA) and enolase (ENO) were upregulated; and one DEG encoding pyruvate kinase (PK) was downregulated (Figure 4 and Table S3). Genes encoding enzymes related to the hydrolysis of polysaccharides into glucose were mostly upregulated. Four DEGs encoding β-glucosidase (β-GC) and two encoding endoglucanase (EG) were upregulated. Similarly, among the four DEGs encoding β-1,3-glucanase (PG), three were upregulated while only one was downregulated (Figure 4 and Table S3). In addition, the expression of *TPP* was significantly reduced in *OsHY5L2*-OE rice grains (Figure 4 and Table S3).

To test the reliability of our transcriptomic analysis, nine DEGs related to starch and sucrose metabolism were randomly selected for qRT-PCR validation. Overall, the qRT-PCR results were consistent with the RNA-Seq results (Figure S3), demonstrating that the transcriptomic data were credible and suitable for further analysis.

### 2.5. Ultrastructure of Starch Granules

**Figure 5:**
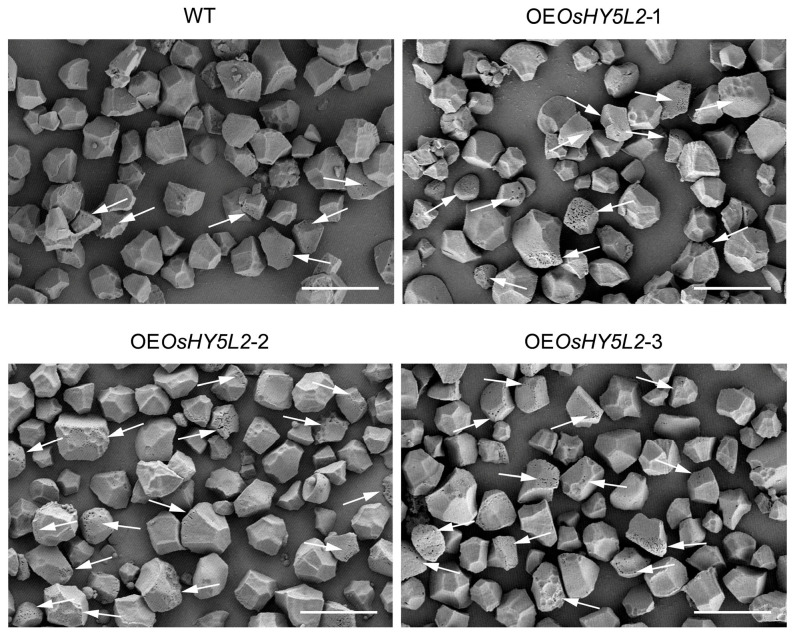
Typical scanning electron micrographs (SEM) of starch granules from *OsHY5L2*-overexpressing lines (OE*OsHY5L2*-1, OE*OsHY5L2*-2, and OE*OsHY5L2*-3) and wild-type (WT) Nipponbare. White arrows indicate pinholes on the surfaces of starch granules. Scale bar = 10 µm.

The morphology and structure of starch granules are key determinants of rice grain quality. Therefore, we examined the ultrastructure of starch granules isolated from the mature grains of WT and *OsHY5L2*-OE transgenic plants. As shown in Figure 5, the starch granules of the WT were irregularly polyhedral with smooth surfaces. However, *OsHY5L2* overexpression resulted in numerous pinholes appearing on the surfaces of the starch granules.

### 2.6. Chain Length Distribution of Amylopectin

**Figure 6:**
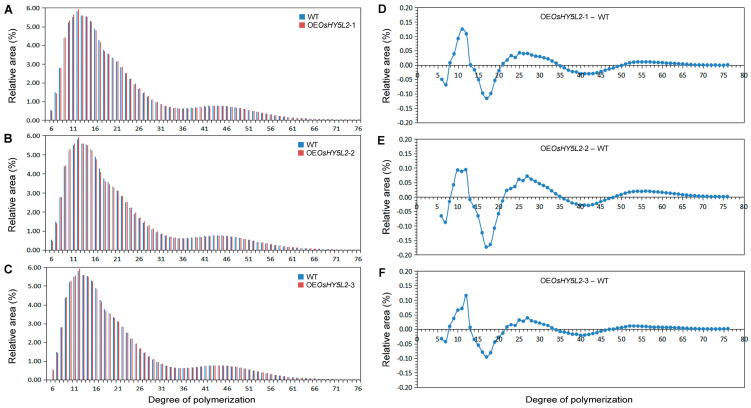
Amylopectin chain length distributions of *OsHY5L2*-overexpressing lines (OE*OsHY5L2*-1, OE*OsHY5L2*-2, and OE*OsHY5L2*-3) and wild-type (WT) Nipponbare. (**A**–**C**) Amylopectin chain length distributions of starch isolated from three transgenic lines and WT after normalization to the total peak area. (**D**–**F**) Differences in the chain length distributions between the transgenic lines and the WT were calculated as follows: the normalized chain length distribution value for each transgenic line minus the value obtained for the WT.

Overexpression of *OsHY5L2* did not significantly alter the average chain length (ACL) or the proportions of A (degree of polymerization, DP 6–12) or B3 (DP ≥ 37) chains in amylopectin (Table 1). However, overexpression of this gene dramatically decreased the proportion of B1 chains (DP 13–24) and significantly increased the proportion of B2 chains (DP 25–36) (Figure 6 and Table 1). Interestingly, although the proportion of A chains (DP 6–12) remained unaffected, the proportion of ultra-short chains (DP 6–7) significantly decreased, and the proportion of short chains (DP 8–12) markedly increased (Figure 6 and Table 1).

### 2.7. Crystalline Structure and Pasting Properties

**Figure 7:**
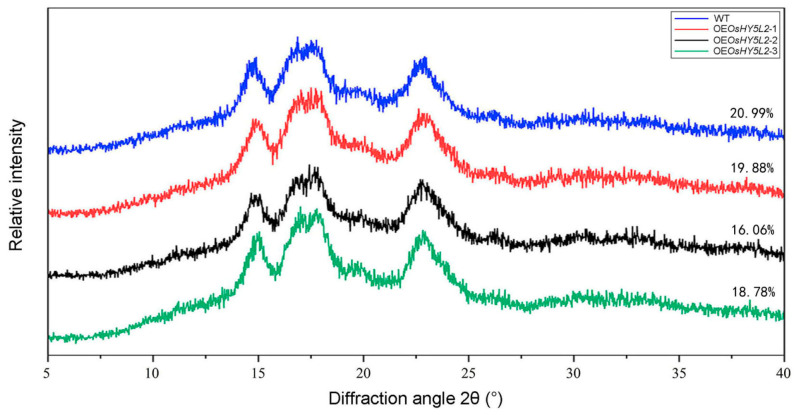
X-ray diffraction (XRD) spectra of starch derived from *OsHY5L2*-overexpressing lines (OE*OsHY5L2*-1, OE*OsHY5L2*-2, and OE*OsHY5L2*-3) and wild-type (WT) Nipponbare.

The starch samples from both WT and *OsHY5L2*-OE lines exhibited typical A-type diffraction patterns with two strong 2θ peaks at about 15° and 23° and an unresolved double 2θ peak at around 17° and 18° (Figure 7), suggesting that *OsHY5L2* overexpression did not alter the A-type crystal pattern of rice starch. However, the relative crystallinities calculated from the XRD patterns differed among the samples. Specifically, starch samples from *OsHY5L2*-OE plants exhibited significantly lower crystallinities than those from WT Nipponbare plants (Figure 7). These results indicated that the overexpression of *OsHY5L2* did not change the crystal structure of rice starch but did affect the stability of rice starch crystals.

Overexpression of *OsHY5L2* significantly affected the pasting properties of starch. Compared with WT Nipponbare rice, overexpression of *OsHY5L2* resulted in a significant increase in the trough viscosity (TV), final viscosity (FV), and setback viscosity (SBV), as well as a notable decrease in the breakdown viscosity (BDV), although it had no significant impact on peak viscosity (PKV) (Table 2). Since the characteristic values of the RVA profile of starch are closely associated with the ECQ of rice, it was suggested that overexpression of *OsHY5L2* has a profound impact on the ECQ of rice.

## 3. Discussion

### 3.1. OsHY5L2 Plays a Positive Regulatory Role in Improving the Appearance and Processing Quality of Rice

Rice chalkiness is not only hereditary but is also influenced by environmental factors such as light and temperature. The physiological causes of chalkiness include an insufficient supply of carbon assimilates produced by leaves and the degradation of starch by α-amylase during grain filling [28,29]. Previous studies have reported that low light stress significantly increases both the chalky grain rate and degree of chalkiness [11,12]. These changes occur due to the reduced net photosynthetic rate of leaves under low-light conditions, leading to an insufficient supply of photosynthetic products to the developing grains [30]. In this study, it was found that *OsHY5L2* overexpression significantly decreased both the chalky grain rate and degree of chalkiness. Because *OsHY5L2* is a positive regulator in the light signaling pathway, it is speculated that the overexpression of *OsHY5L2* may inhibit the formation of chalky rice grains by increasing the supply of photosynthetic assimilates. Accelerated starch biosynthesis due to the increased activities of starch biosynthesis-related enzymes in developing rice grains is also an important cause of chalkiness formation. It has been previously reported that the activities of AGPase and SBE in a near-isogenic line CSSL50-1 with high chalkiness were higher than those in the normal parental line Asominori at days 15 and 20 after flowering [31]. In this study, we found that the activities of these two enzymes were significantly decreased (Figure 2A,D). This decrease is perhaps one of the reasons why the formation of chalky grains was significantly inhibited in the *OsHY5L2*-OE lines.

Interestingly, although *OsHY5L2* overexpression substantially increased the head rice rate, it did not significantly impact the brown rice rate or milled rice rate, indicating that overexpression of *OsHY5L2* did not affect the development of the glume and pericarp of the rice seeds. The processing quality and appearance quality of rice are closely related. Excessive rice chalkiness will lead to a reduced head rice rate because the chalky endosperm comprises loosely packed starch granules with large air spaces and is thus less stable and more prone to breakage during milling [32]. Therefore, overexpression of *OsHY5L2* increased the head rice rate of transgenic lines by inhibiting the occurrence of chalkiness in rice grains.

### 3.2. OsHY5L2 Negatively Regulates the Accumulation of Starch in Rice Endosperm by Inhibiting Starch Biosynthesis and Promoting Starch Hydrolysis

Starch metabolism is critical for the content and composition of starch in rice endosperm, thereby influencing rice quality. Overexpression of *OsHY5L2* was found to significantly decrease the total starch content and AAC (Figure 1D,E). AGPase carries out the first key step of starch biosynthesis and is the most important rate-limiting enzyme [33]. We speculate that the reduced total starch content of *OsHY5L2*-OE rice grains was the result of reduced AGPase activity. GBSS is responsible for the biosynthesis of amylose [34]. The significantly reduced activities of GBSS in *OsHY5L2*-OE grains may reasonably explain the observed decreases in the AAC. When sucrose is transported from sources (i.e., leaves) to sinks (i.e., seeds), it is first converted into UDP-glucose (UDPG) via sucrose synthase (SS). UDPG is converted to ADPG by UDPG pyrophosphorylase (UGP) and ADPG pyrophosphorylase (AGPase), which is subsequently used to synthesize starch [35]. Here, we observed that overexpression of *OsHY5L2* resulted in the upregulated expression of the gene encoding INV but downregulated expression of the gene encoding SS (Figure 4). These results suggest that the conversion of sucrose into glucose is enhanced in *OsHY5L2*-OE grains, although the ability to generate substrates for starch biosynthesis is weakened. Coupled with the enzyme activity results (Figure 2), it appears that *OsHY5L2* regulates starch biosynthesis in rice endosperm by inhibiting the generation of UDPG and thus decreasing the activity of starch biosynthesis-related enzymes such as AGPase and GBSS.

Starch accumulation is regulated by the balance between starch biosynthesis and degradation. The phytohormones gibberellins (GAs) and abscisic acid (ABA) regulate starch hydrolysis in cereal endosperm. GAs promote starch hydrolysis by increasing the activity of α-AMY, whereas ABA acts antagonistically [36]. In this study, we observed that overexpression of *OsHY5L2* resulted in a significant increase in GA_1_ levels and the activities of both α-AMY and β-AMY in *OsHY5L2*-OE grains, while the levels of ABA remained unchanged (Figure 3A–C,F). Transcriptomic data also showed that the expression of genes encoding α-AMY was significantly up-regulated in the *OsHY5L2*-OE grains (Figure 4). These results suggest that *OsHY5L2* positively regulates starch hydrolysis by inducing GA_1_ biosynthesis. Our findings are consistent with previous studies which demonstrated that SlHY5 mediates blue light-induced starch hydrolysis in tomato by activating starch hydrolysis-related genes such as *PWD*, *BAM1*, *BAM3*, *BAM8*, *MEX1*, and *DPE1* [37]. Therefore, considering that overexpression of *OsHY5L2* decreased the activities of most of enzymes involved in starch biosynthesis, it was concluded that *OsHY5L2* regulates the accumulation of starch in rice endosperm by inhibiting starch biosynthesis and promoting starch hydrolysis.

Accumulating evidence suggests that high temperature stress increases α-AMY activity in the grains of rice and wheat, leading to the formation of pinhole structures on the surfaces of their starch granules [38,39]. Nakata et al. [40] provided direct evidence that increased α-AMY activity alters starch morphology by demonstrating that overexpression of genes encoding α-AMY results in the formation of pinhole structures on the surface of rice endosperm starch granules [40]. Our results showed that overexpression of *OsHY5L2* significantly increased the expression of genes encoding α-AMY, as well as the activity of α-AMY (Figure 3A and Figure 4), suggesting that α-AMY-mediated starch hydrolysis may underlie the formation of pinhole structures on the surfaces of starch granules in *OsHY5L2*-OE rice grains.

### 3.3. Overexpression of OsHY5L2 Alters the Fine Structure and Physicochemical Properties of Endosperm Starch

It was reported that starches from germinated rice grains tend to exhibit decreased proportions of short and intermediate chains but increased proportions of long-chain amylopectin branches [41,42]. Similarly, *OsHY5L2* overexpression promotes starch hydrolysis and decreases the proportion of intermediate chains, but also increases the proportion of long chains of amylopectin (Figure 3 and Figure 6 and Table 1). These findings suggest that *OsHY5L2* regulates the chain length distribution of amylopectin by promoting starch hydrolysis. The crystallinity of rice starch is closely associated with the AC and chain length distribution of amylopectin. AC is generally negatively associated with crystallinity. Higher amylose contents result in lower crystallinity due to the majority of amylose molecules being present in the amorphous regions of starch granules [43]. Overexpression of *OsHY5L2* significantly reduced both the AAC and crystallinity (Figure 1E and Figure 7), indicating that the reduced crystallinity observed in OsHY5L2-OE lines was likely not attributable directly to the AC. Vandeputte and Delcour [44] reported that the crystalline characteristics of starch are determined by the formation of double helical structures between adjacent A and B chains within the same cluster in the crystalline lamellae. The formation and regular arrangement of these double helices require specific A and B chain lengths [44]. Jane et al. [45] reported that the crystalline lamellae are predominantly composed of amylopectin chains with DP 15–20, and that chains with DP 18–21 can span the entire crystalline lamellae, indicating a positive association between the proportion of intermediate amylopectin chains and crystallinity. In our study, we observed that *OsHY5L2* overexpression significantly reduced the proportion of intermediate chains of amylopectin (Figure 6 and Table 1). Therefore, it was speculated that the altered starch crystallinity of the *OsHY5L2*-OE lines may have resulted from a change in the proportion of intermediate chains of amylopectin. On the other hand, the relative crystallinities of starches from germinated rice grains were found to be significantly lower than those from non-germinated samples [46]. Thus, another possible explanation may be the increased hydrolysis of endosperm starch in *OsHY5L2*-OE lines.

It is generally believed that rice varieties with better cooking quality exhibit high BDVs and low SBVs [47]. The decreased BDV and increased SBV of starch from *OsHY5L2*-OE lines suggest that the *OsHY5L2*-OE grains may exhibit relatively poor cooking quality. Han and Hamaker [48] found that the proportion of long chains of amylopectin was negatively correlated with BDV, while the proportion of short chains was positively correlated with BDV. A high proportion of short chains increases the amount of amorphous regions in starch granules, causing the starch to gelatinize more readily, thereby increasing the BDV. Conversely, a higher proportion of long chains strengthens intermolecular interactions and stabilizes the structure of amylopectin, decreasing BDV [49]. Chen et al. [50] demonstrated that in rice varieties with lower gelatinization temperatures (Longtefu B), knockdown of the *ALK* gene (encoding SSII-3) increased the BDV while reducing the SBV. Furthermore, knockdown of the *ALK* gene increased the proportion of chains with DP 6–8 and DP 13–30 but reduced the proportion of chains with DP 9–12. This result indicated that the proportion of chains with DP 6–8 and DP 13–30 is positively associated with BDV but negatively associated with SBV. In addition, the proportion of chains with DP 9–12 was negatively associated with BDV but positively associated with SBV [50]. Consistent with these results, we observed that overexpression of *OsHY5L2* reduced the proportions of ultra-short chains (DP 6–7) and intermediate chains (DP 13–24) of amylopectin, while increasing the proportions of short chains (DP 8–12) and long chains (DP 25–36) (Figure 6 and Table 1). Moreover, *OsHY5L2* overexpression significantly decreased the BDV and increased the SBV of rice starch (Table 2). Together, these findings suggest that *OsHY5L2* influences the pasting properties of endosperm starch by altering the chain length distribution of amylopectin.

Although some meaningful results were obtained in our study, there remain some limitations. First, the knockout or knockdown of *OsHY5L2* caused seedling lethality in rice [27], making it impossible to evaluate the effects of *OsHY5L2* mutation on starch structure and physicochemical properties. Second, RNA-seq was performed on only a single *OsHY5L2*-OE line in our study, limiting generalization. Third, while *OsHY5L2* functions as a bZIP transcription factor essential for light signaling, its direct target genes remain unknown. Therefore, the potential pleiotropic effects of *OsHY5L2* on other physiological processes could not be completely excluded. Future research should focus on unraveling the molecular mechanisms of light signaling in rice quality regulation and defining the specific regulatory role of *OsHY5L2* in this process.

## 4. Materials and Methods

### 4.1. Plant Materials and Growth Conditions

The collection of plant material was performed in accordance with the relevant institutional, national, and international guidelines and legislation. Wild-type rice (*Oryza sativa* cv Nipponbare) and three *OsHY5L2* overexpression lines were used. The seeds of wild-type Nipponbare were obtained from BioRun Biosciences Co., Ltd. in Wuhan, Hubei Province, China. The *OsHY5L2*-overexpressing lines were constructed in our laboratory at Jiangxi Agricultural University (JXAU). All experiments were conducted at the Key Laboratory of Crop Physiology of JXAU of Nanchang City, Jiangxi Province, China. Pre-germinated rice seeds were sown in wet paddy soils. After approximately three weeks, the seedlings were transplanted into plastic pots (27.5 cm in length, 21.0 cm in width and 32.0 cm in height) containing 10 kg of soil. Each pot contained two hills, and each hill contained two seedlings. Prior to transplanting, each pot was supplemented with 1.625 g of urea, 1.75 g of potassium chloride, and 6.25 g of calcium-magnesium phosphate. At the tillering stage, an additional 0.625 g of urea was added to each pot. During the panicle initiation stage, each pot received an additional 1.0 g of urea and 0.75 g of potassium chloride. After transplanting, the seedlings were transferred to environmentally controlled chambers (30 °C day/25 °C night, 12 h light/12 h dark photoperiod, 800 μmol m^−2^ s^−1^ light intensity) and allowed to grow until maturity. At least 40 plants from each transgenic line or wild-type were used for each experiment, and three independent experiments were performed.

### 4.2. Gene Cloning and Transformation

To generate the *OsHY5L2* overexpressing transgenic lines, the coding sequence (CDS) of *OsHY5L2* (Os06g39960) was cloned from rice (*Oryza sativa* cv. Nipponbare). The amplified *OsHY5L2* CDS was inserted into the pBWA(V)HS-GFP vector to construct the *35S::OsHY5L2* fusion gene. Subsequently, the construct was introduced into the *Agrobacterium tumefaciens* strain GV3101. Rice plants were transformed according to the method of Hiei et al. [51]. In total, nine independent *OsHY5L2* transgenic lines were obtained. PCR analysis showed that the *OsHY5L2* gene was integrated successfully into the genomes of the transformed rice plants (Figure S4A). The expression levels of *OsHY5L2* in nine independent transgenic lines (OE*OsHY5L2*-1–9) were verified using qRT-PCR. As expected, all transgenic lines exhibited higher relative expression levels of *OsHY5L2* than the WT (Figure S4B). Consistent with a previous report [27], all the transgenic lines exhibited photomorphogenesis-related phenotypes such as dwarfism, deep green leaves, and shortened internodes, indicating that the phenotypes of *OsHY5L2*-OE transgenic lines were caused by increased expression levels of *OsHY5L2*, not by positional insertion. Three independently transgenic lines (OE*OsHY5L2*-1, OE*OsHY5L2*-2, and OE*OsHY5L2*-3) with higher expression levels were then selected for further study.

### 4.3. Transcriptomic Sequencing and Analysis

To ascertain which pathways might be regulated by OsHY5L2, RNA-Seq analysis was conducted to identify the genes that were differentially expressed in wild-type and *OsHY5L2*-OE plants. RNA extraction, library construction, and sequencing were performed by BioMarker Technologies Corporation (Beijing, China). Briefly, RNA was extracted from the developing grains (two weeks post-anthesis) of the wild-type (WT) and OE*OsHY5L2*-1 line using an EASYspin Plant RNA Extraction Kit (Aidlab, Beijing, China). cDNA libraries were constructed and sequenced on an Illumina platform for high-throughput analysis. Raw reads were filtered to remove reads containing adapter sequences and reads of low quality. All downstream analyses utilized only high-quality clean reads. Differentially expressed genes (DEGs) were selected using the DESeq2 R package (version 1.42.1), according to the criteria of a |Log_2_ fold change (FC)| ≥ 1 and adjusted false discovery rate (FDR) < 0.01. GO enrichment analysis of the DEGs was conducted using the GOseq R packages (version 1.40.0). The KOBAS 2.0 software was used to determine the statistical enrichment of DEGs in the KEGG pathways [52]. A *p* value < 0.05 was also the threshold for DEGs in the GO terms and KEGG pathways.

### 4.4. qRT-PCR Validation of DEGs

Quantitative real-time PCR (qRT-PCR) was used to validate the reliability of the transcriptomic data. Total RNA was extracted from the developing grains (two weeks post-anthesis) of WT and transgenic *OsHY5L2*-OE rice at the grain-filling stage using a TRIzol reagent. First-strand cDNA was synthesized using the GoScript™ Reverse Transcription System following the manufacturer’s instructions. qRT-PCR was performed using a CFX96 Real-Time PCR System (Bio-Rad, Hercules, CA, USA) with Hieff ^®^ qPCR SYBR ^®^ Green Master Mix (Yeasen, Shanghai, China). The thermal cycling conditions included initial denaturation at 95 °C for 2 min, followed by 40 cycles of 95 °C for 10 s and 60 °C for 30 s. Gene expression levels were calculated via the 2^−Δcq^ method described by Bio-Rad (http://www.bio-rad.com/zh-cn/applications-technologies/real-time-pcr-experimental-design) (accessed on 15 June 2024). All gene-specific primers were designed based on the cDNA sequences (Table S4). The rice ubiquitin gene (*OsUBI*, Os03g13170) was used as an internal reference.

### 4.5. Evaluation of Starch-Metabolism-Related Physiological Parameters

To investigate the effects of *OsHY5L2* overexpression on the starch metabolism in developing rice grains, the starch-metabolism-related physiological parameters were also examined. The contents of endogenous phytohormones in the developing grains (two weeks post-anthesis) of WT and transgenic *OsHY5L2*-OE rice were measured using ultrahigh-performance liquid chromatography–tandem mass spectrometry (UPLC-MS/MS, Thermo Fisher Scientific, Waltham, MA, USA). Briefly, approximately 100 mg of each sample was extracted in 1 mL of a 50% aqueous acetonitrile solution. Following ultrasonication, the samples were further extracted using a Stuart SB3 benchtop laboratory rotator. Then, the samples were centrifuged, and the supernatant was purified. UPLC was performed using a UPLC Orbitrap-MS system. The activities of AGPase (ADP-glucose pyrophosphorylase, M1107A), SSS (soluble starch synthase, M1108A), GBSS (granule-bound starch synthase, M1109A), SBE (starch branching enzyme, M1110A), DBE (starch debranching enzyme, M1111A), SP (sucrose phosphorylase, M1112A), α-AMY (α-amylase, M1104A), and β-AMY (β-amylase, M1105A), as well as the contents of starch (M1101A) and glucose (M1501A), were measured using commercial kits following the manufacturer’s instructions (Suzhou Michy Biomedical Technology Co., Ltd., Suzhou, China).

### 4.6. Evaluation of Rice Processing Quality and Appearance

The samples (250 g each) stored for more than three months were dehulled using a sheller (THU35C, SATAKE, Higashihiroshima, Japan) and milled using a rice miller (TM05C, SATAKE, Higashihiroshima, Japan). The head rice chalky grain rate and chalkiness degree were measured using an MRS-9600TFU2L rice appearance quality tester (MICROTEK, Shenzhen, China). The brown rice rate, milled rice rate, head rice rate, chalky grain rate, and chalkiness degree were calculated as follows:

Brown rice rate (%) = (brown rice weight/paddy weight) × 100

Milled rice rate (%) = (milled rice weight/paddy weight) × 100

Head rice rate (%) = (head rice weight/paddy weight) × 100

Chalky grain rate (%) = number of chalky grains/number of observed grains × 100

Chalkiness degree (%) = chalky grain rate × chalky area rate × 100

### 4.7. Starch Extraction and Scanning Electron Microscopy

The polished rice grains (10 g) were soaked in deionized water and held overnight at room temperature. The soaked grains were milled with iced distilled water using a kitchen blender (MJ-BL1206A, Foshan, China), and the slurry was centrifuged. Then, the precipitate was resuspended and centrifuged. This procedure was repeated using anhydrous ethanol followed by distilled water three times. Next, 1.6 g/L protease (Amresco, Solon, OH, USA) was added, and the mixture was stirred with a magnetic stirring bar for 2 h at 37 °C. The slurry was then centrifuged, and the whitish precipitate was mixed with 2.5 g/L NaOH solution, followed by further centrifugation. The precipitated starch was desiccated and passed through a 100-mesh filter.

In preparation for scanning electron microscopy (SEM), starch samples were dispersed in anhydrous ethanol. The solution (2 μL) was then dropped onto aluminum foil films and stored at 40 °C for 5 h. Subsequently, starch samples were coated with gold, observed, and photographed using field emission SEM (Zeiss Merlin Compact, Oberkochen, Germany).

### 4.8. Analysis of Amylopectin Chain-Length Distribution

Amylopectin chain length distribution was determined with high-performance anion-exchange chromatography (HPAEC). Approximately 20 mg of each starch sample was resuspended in 10 mL of water and heated for 1 h. Then, 50 μL of sodium acetate buffer, 10 μL of sodium azide solution, and 10 μL of isoamylase solution were added to each sample. The mixtures were incubated at 37 °C for 24 h. Subsequently, 0.5% sodium borohydride solution was added, and the samples were allowed to stand for 20 h. The samples were then centrifuged, and the supernatants were collected for further analysis. The chain length distributions were analyzed using a Thermo ICS5000+ system (Thermo Fisher Scientific, Waltham, MA, USA).

### 4.9. X-Ray Diffraction Analysis

Analysis of the XRD patterns of starch was performed using a Dmax-2200PC X-ray diffractometer (Rigaku Corporation, Akishima, Japan) operated at 40 kV and 40 mA with Cu-Kα radiation. The scattering angle (2θ) was scanned from 5° to 40° at a scanning rate of 0.02°/min. The degree of crystallinity was calculated as X_c_ = A_c_/(A_c_ + A_a_), where X_c_ is the degree of crystallinity, and A_c_ and A_a_ are the areas of the crystalline peak and amorphous peak, respectively.

### 4.10. Measurement of Pasting Properties

Starch pasting properties were determined using a Rapid Visco Analyzer (Super 4 RVA, Newport Scientific, Highett, Australia) according to a previously published method [53]. The starch sample (passed through a 100-mesh screen, 2 g) was mixed with deionized water (25 mL) in an aluminum pan. The pasting program cycle was set to 13 min. Starch samples started at 50 °C for 1 min and were then heated from 50 °C to 95 °C at 12 °C/min, held at 95 °C for 2 min, cooled to 50 °C at 12 °C/min, and held again for 2 min. Data on the pasting properties were collected and analyzed with the Thermal Cycle for Windows (TCW3) software. The following starch pasting parameters were evaluated: peak viscosity (PKV), through viscosity (TV), final viscosity (FV), breakdown viscosity (BDV = PKV − TV), and setback viscosity (SBV = FV − PKV).

### 4.11. Data Analysis

All experiments were performed with three biological replicates. Data represent the mean ± SD (standard deviation) with three biological replicates (each with three technical repeats) for WT and individual transgenic lines. A one-way Analysis of Variance (ANOVA) was used to determine statistically significant differences in means, and an ANOVA and Tukey’s test was calculated when statistical differences were observed using the SPSS 25.0 statistical software (IBM, Armonk, NY, USA). Results with *p* < 0.05 were considered statistically significant.

## 5. Conclusions

Light is one of the most important environmental factors that affects crop productivity and quality. Numerous experimental data and related reports have confirmed that low light dramatically affects the physiological traits of rice plants, leading to reduced grain quality. However, the functions of key genes in the light signaling pathway in terms of rice quality regulation remain largely unexplored. In this study, we found that *OsHY5L2*, encoding a central component in the light signaling pathway, plays an important role in regulating the metabolism, fine structure, and physicochemical properties of rice endosperm starch. *OsHY5L2* appears to play a positive role in determining rice processing quality and appearance. Overexpression of *OsHY5L2* alters the accumulation of starch in rice endosperm by inhibiting starch biosynthesis and promoting starch hydrolysis. These alterations to starch metabolism resulted in profound changes in the morphology of starch granules, including the formation of pinhole structures on the surfaces of the starch granules. Furthermore, *OsHY5L2* overexpression altered the physicochemical properties of rice starch by affecting its multi-level structure and function. These changes were attributed to decreased proportions of ultra-short chains and intermediate chains of amylopectin and an increased proportion of long chains of amylopectin. Our findings hold significant importance for understanding the molecular mechanisms of light-regulated rice quality and for breeding high-quality rice varieties that can adapt to changes in the environment.

## Figures and Tables

**Table 1:** Amylopectin chain length distributions of starch isolated from *OsHY5L2*-overexpressing lines (OE*OsHY5L2*-1, OE*OsHY5L2*-2, and OE*OsHY5L2*-3) and wild-type (WT) Nipponbare.

Samples	A (%)			B1 (%)	B2 (%)	B3 (%)	ACL (DP)
	DP6–7	DP8–12	DP6–12	DP13–24	DP25–36	DP ≥ 37	
WT	2.06 ± 0.03 ^a^	23.74 ± 0.12 ^b^	25.80 ± 0.15 ^a^	47.04 ± 0.08 ^a^	12.57 ± 0.05 ^c^	14.59 ± 0.05 ^a^	21.45 ± 0.12 ^a^
OE*OsHY5L2*-1	1.94 ± 0.05 ^bc^	24.11 ± 0.12 ^a^	26.05 ± 0.17 ^a^	46.62 ± 0.07 ^b^	12.90 ± 0.04 ^b^	14.43 ± 0.13 ^a^	21.45 ± 0.05 ^a^
OE*OsHY5L2*-2	1.91 ± 0.01 ^c^	24.04 ± 0.04 ^a^	25.95 ± 0.03 ^a^	46.37 ± 0.02 ^c^	13.04 ± 0.01 ^a^	14.64 ± 0.02 ^a^	21.56 ± 0.01 ^a^
OE*OsHY5L2*-3	1.96 ± 0.03 ^b^	24.04 ± 0.09 ^a^	26.00 ± 0.12 ^a^	46.58 ± 0.11 ^b^	12.85 ± 0.03 ^b^	14.57 ± 0.06 ^a^	21.47 ± 0.02 ^a^

The values in the same column with different letters differ significantly at *p* < 0.05, based on Tukey’s test.

**Table 2:** Pasting properties of starch from *OsHY5L2*-overexpressing lines (OE*OsHY5L2*-1, OE*OsHY5L2*-2, and OE*OsHY5L2*-3) and wild-type (WT) Nipponbare, as measured with RVA.

Samples	PKV (cP)	TV (cP)	FV (cP)	BDV (cP)	SBV (cP)
WT	1379 ± 18 ^a^	818 ± 10 ^b^	1427 ± 16 ^b^	561 ± 10 ^a^	48 ± 7 ^c^
OE*OsHY5L2*-1	1384 ± 26 ^a^	885 ± 15 ^a^	1547 ± 23 ^a^	499 ± 11 ^b^	163 ± 6 ^b^
OE*OsHY5L2*-2	1364 ± 16 ^a^	892 ± 14 ^a^	1563 ± 18 ^a^	472 ± 10 ^c^	199 ± 4 ^a^
OE*OsHY5L2*-3	1363 ± 10 ^a^	885 ± 10 ^a^	1553 ± 20 ^a^	478 ± 5 ^c^	190 ± 11 ^a^

The values in the same column with different letters differ significantly at *p* < 0.05, based on Tukey’s test.

## Data Availability

The original contributions presented in this study are included in the article/Supplementary Materials. Further inquiries can be directed to the corresponding authors.

## Supplementary Materials

The following supporting information can be downloaded at: https://www.mdpi.com/article/10.3390/plants14182888/s1. Figure S1: GO classification of biological processes, cellular components, and molecular functions associated with DEGs from *OsHY5L2*-overexpressing line (OE*OsHY5L2*-1) and wild-type (WT) Nipponbare; Figure S2: KEGG pathway enrichment analysis of DEGs from *OsHY5L2*-overexpressing line (OE*OsHY5L2*-1) and wild-type (WT) Nipponbare; Figure S3: qRT-PCR analysis of the expression levels of *INV*, *SS*, *α-AMY*, *β-AMY*, *PFK*, *β-GC*, *EG*, *PG*, and *TPP* in three *OsHY5L2* transgenic lines (OE*OsHY5L2*-1, OE*OsHY5L2*-2, and OE*OsHY5L2*-3) and WT Nipponbare. The expression levels of each gene are shown as a ratio relative to the control. Error bars = SD. Mean value followed by different letters are significantly different (*p* < 0.05); Figure S4: Molecular identification of *OsHY5L2*-overexpressing transgenic lines. (A) PCR patterns of *OsHY5L2* transgenic lines (OE*OsHY5L2*-1–9) and wild-type (WT) Nipponbare utilizing primers specific to the *35S::OsHY5L2* fusion gene. Marker, DL2,000 DNA marker. (B) qRT-PCR validation of the relative transcription levels of *OsHY5L2* in *OsHY5L2* transgenic lines. Error bars = SD. Mean value followed by different letters are significantly different (*p* < 0.05); Table S1: List of up-regulated DEGs identified in OEOsHY5L2-1 vs. the WT; Table S2: List of down-regulated DEGs identified in OEOsHY5L2-1 vs. the WT; Table S3: List of representative DEGs related to starch and sucrose metabolism. Table S4: The list of primers used in this study.
