# Supplementary material for: Overexpression of the OsHY5L2 Alters the Fine Structure and Physicochemical Properties of Endosperm Starch in Rice (Oryza sativa L.)
Source: Plants (Basel). 2025 Sep 17;14(18):2888. doi: 10.3390/plants14182888 (PMC12473966; doi:10.3390/plants14182888)
Supplement: Supplementary file 1 [file plants-14-02888-s001.zip › plants-3830381-supplementary/Supplementary Files/Supplementary Figures.pdf]

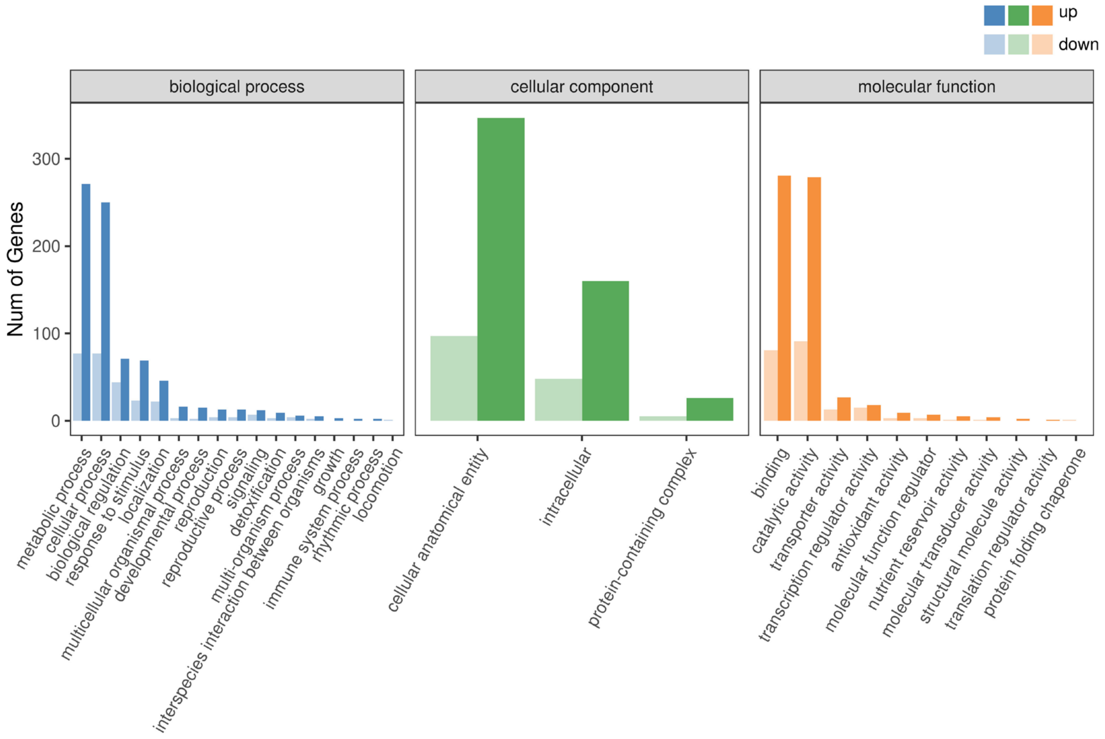

Figure S1. GO classification of biological processes, cellular components, and molecular functions associated with DEGs from *OsHY5L2*-overexpressing line (OE*OsHY5L2*-1) and wild-type (WT) Nipponbare.

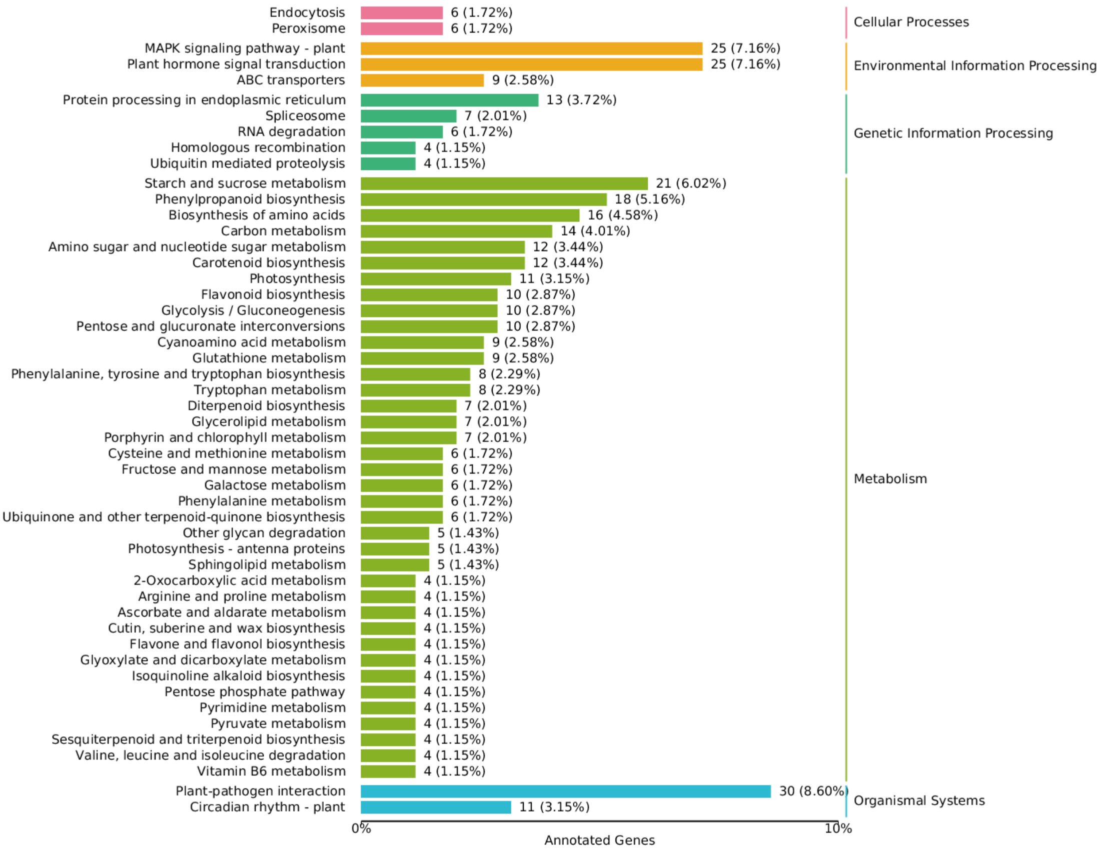

Figure S2. KEGG pathway enrichment analysis of DEGs from *OsHY5L2*-overexpressing line (OE*OsHY5L2*-1) and wild-type (WT) Nipponbare.

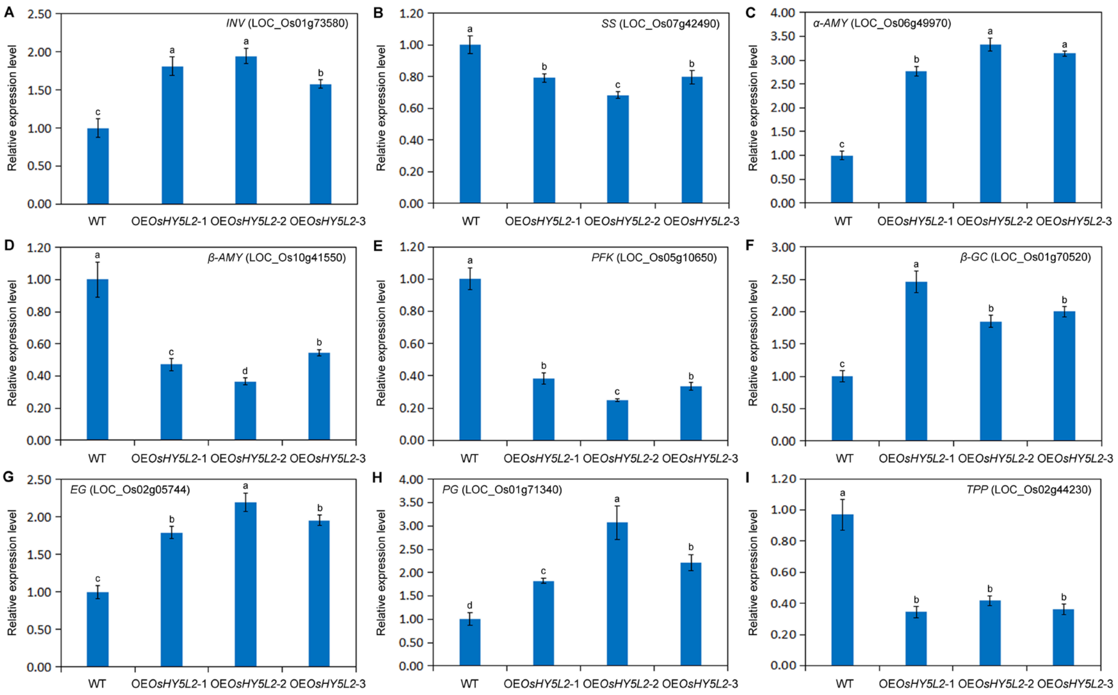

Figure S3. qRT-PCR analysis of the expression levels of *INV*, *SS*, *α-AMY*, *β-AMY*, *PFK*, *β-GC*, *EG*, *PG*, and *TPP* in three *OsHY5L2* transgenic lines (OE*OsHY5L2*-1, OE*OsHY5L2*-2, and OE*OsHY5L2*-3) and WT Nipponbare. The expression levels of each gene are shown as a ratio relative to the control. Error bars = SD. Mean value followed by different letters are significantly different ($P < 0.05$).

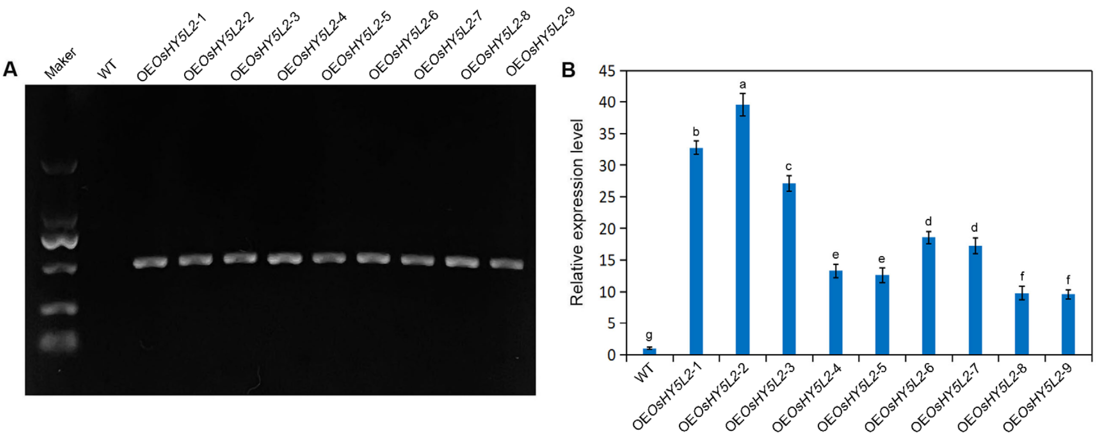

Figure S4. Molecular identification of *OsHY5L2*-overexpressing transgenic lines. (A) PCR patterns of *OsHY5L2* transgenic lines (OE*OsHY5L2*-1–9) and wild-type (WT) Nipponbare utilizing primers specific to the *35S::OsHY5L2* fusion gene. Marker, DL2,000 DNA marker. (B) qRT-PCR validation of the relative transcription levels of *OsHY5L2* in *OsHY5L2* transgenic lines. Error bars = SD. Mean value followed by different letters are significantly different ($P < 0.05$).
